# New Remedies

**Published:** 1884-06

**Authors:** Wm. A. Pease


### New Remedies.

BY DR. WM. A. PEASE.

A paper appeared in the January number of the DENTAL REGISTER, 1882, which I read before the Mad River Dental Society, entitled, "A New Departure." That paper grew out of the unsatisfactory results in the treatment of teeth from which the pulps had been more or less perfectly removed; and of that chronic, irritable condition, often cystic, which surrounded the apices of the roots. And of numerous experiments, observation and failures which had convinced me that the most thorough antiseptic treatment of dead pulps, or remnants of them, in some mouths did not antisept. That after a time decomposition would be resumed, and gas and irritation would be the result. Still there was a class of persons of such robustitude; so little susceptible to irritation; whose absorbents were so active and healthy; in the sockets of whose teeth dead pulps created little or no irritation; that it made the antiseptic theory seem plausible, while failures in the numerous teeth of irritable patients were regarded as exceptional--due to improper treatment. These cases were too numerous to be satisfactory to me, and I began to regard the treatment as unphilosophical, and then to experiment to see to what extent the antiseptic treatment was successful. Pulps deadened with arsenic, fully exposed, and covered with carbolic acid, and then covered with a filling, soon gave trouble. Others, treated in the same way with wood creosote, were equally troublesome, and others punctured with a broach, to enable the carbolic acid or creosote to be absorbed, ultimately gave trouble. Pieces of the best salted and smoked meat, bathed with carbolic acid or creosote, when subjected to moisture and the temperature of the blood, soon decomposed. These experiments, and others, were repeated in various forms until I became convinced the antiseptic treatment did not antisept. In the presence of these facts what was to be done? Remove the pulp as far as possible? That was already the practice; but it was often incomplete in the curved roots of the molars, and there were often fragments left in the single rooted teeth. Under the old theory the remains of the pulp were supposed to oxydize—a slow process of cremation; under the new, they are attacked by living organisms, which hasten metamorphosis by their individual action. The results of the processes are the same; but they have a different initiation. Now it is believed that carbolic acid destroys these organisms; and probably it does, and the putrefactive process is held in abeyance for a time, to be revived by a succeeding visitation. Just how that visitation finds ingress to a plugged tooth, passes some sort of an impediment, more or less impregnated with carbolic acid, to reach a fragment of partially carbolized pulp, is not easily explained. Certainly teeth are extracted that have two distinct stinks; the one from a partially filled root, from which the smell of carbolic acid has not entirely left; the other from putrefying animal matter beyond it. It is supposed some one will find the key to unlock this enigma, but at present we are left to various conjectures.

If my memory serves me, at the time I read that paper, no means for removing or rendering the remains of the pulp inert were proposed ; certainly not the one now used. That paper was tentative. It proposed a theory to be worked out by many experiments by many men. As I have no copy of that paper, and have never seen it in the REGISTER, I am embarrassed now by not knowing what it contained.

At the last meeting of the Mad River Dental Society I had a paper, which was not read, relating to some conditions of the dentine immediately preceding the death of the pulp from natural causes, and after decomposition had set in, which bore upon and threw some light upon this subject, but which is too long to be inserted here. I must be content after another year of experiment and of watching results to record the means I have found the most effective, which are believed to be valuable and novel. They consist of iod. potass et aqua calcis; both strong, the strength to be determined by location, patient, and what is required. Where there is much tissue to be destroyed the action of the agent may be facilitated by probing and lacerating it with broaches and pumping the solution into the substance of it, or by letting it be absorbed by gravitation or capillary attraction. This will be chiefly necessary beyond the curve of the roots of the molars. Several applications may be made when necessary, until the tissue has become saponified, when it can generally be washed out by a syringe being more or less soluble, or by arming a broach with sufficient cotton wet in the solution and then passing it down the canal, twirling it around and removing it. Sometimes a broach made from a splint of the bamboo, which is very tough and flexible, is better. Then, if any remain, it is impregnated with potash, saponified, inert, and a most uncongenial *nidus* for parasites, while the iodine would evaparate and gently stimulate the absorbents beyond the foramen. This method leaves the dentine clean and white, and the passages leading to the crusta petrosa are not dark as when they are infiltrated with carbolic acid. As an adjuvant to them the bromide of potass may be freely used of any strength desirable, as it is the best sedative of irritated mucus membrane and of the nerves, which terminate immediately beneath it, we have.

Here, let me add a postscript, but no way pertinent to the subject, that iodie et bromide potassæ in suitable solution are the best mouth washes for certain conditions we have. Either may be used as indicated. They cleanse the teeth well, and have a salutary effect on the gum. One, or the other of them, will, often after a few applications, subdue the tenderness in cavities near the gum; as well as those unstable and fugitive pains in irritable and hysterical patients; no injury is caused by swallowing a little of either, and for the hysterical a full dose of the bromide is often beneficial.

